# Land Surface Reflectance Retrieval from Hyperspectral Data Collected by an Unmanned Aerial Vehicle over the Baotou Test Site

**DOI:** 10.1371/journal.pone.0066972

**Published:** 2013-06-12

**Authors:** Si-Bo Duan, Zhao-Liang Li, Bo-Hui Tang, Hua Wu, Lingling Ma, Enyu Zhao, Chuanrong Li

**Affiliations:** 1 State Key Laboratory of Resources and Environment Information System, Institute of Geographic Sciences and Natural Resources Research, Chinese Academy of Sciences, Beijing, China; 2 University of Chinese Academy of Sciences, Beijing, China; 3 Laboratoire des sciences de l'ingenieur, de l'informatique et de l'imagerie, Université de Strasbourg, Centre National de la Recherche Scientifique, Illkirch, France; 4 Key Laboratory of Agri-informatics,Ministry of Agriculture/Institute of Agricultural Resources and Regional Planning, Chinese Academy of Agricultural Sciences, Beijing, China; 5 Earth Observation Technology Application Department, Academy of Opto-Electronics, Chinese Academy of Sciences, Beijing, China; University of Western Australia, Australia

## Abstract

To evaluate the in-flight performance of a new hyperspectral sensor onboard an unmanned aerial vehicle (UAV-HYPER), a comprehensive field campaign was conducted over the Baotou test site in China on 3 September 2011. Several portable reference reflectance targets were deployed across the test site. The radiometric performance of the UAV-HYPER sensor was assessed in terms of signal-to-noise ratio (SNR) and the calibration accuracy. The SNR of the different bands of the UAV-HYPER sensor was estimated to be between approximately 5 and 120 over the homogeneous targets, and the linear response of the apparent reflectance ranged from approximately 0.05 to 0.45. The uniform and non-uniform Lambertian land surface reflectance was retrieved and validated using *in situ* measurements, with root mean square error (RMSE) of approximately 0.01–0.07 and relative RMSE of approximately 5%–12%. There were small discrepancies between the retrieved uniform and non-uniform Lambertian land surface reflectance over the homogeneous targets and under low aerosol optical depth (AOD) conditions (AOD = 0.18). However, these discrepancies must be taken into account when adjacent pixels had large land surface reflectance contrast and under high AOD conditions (e.g. AOD = 1.0).

## Introduction

Hyperspectral data in the solar-reflective region (0.4–2.5 µm) has been collected since the mid-1980 s [1]. Hyperspectral remote sensing is increasingly being used in a wide range of applications, including geology, agriculture, forestry, and ecology [2]–[4].

An adequate pre-processing of hyperspectral data is a mandatory prerequisite to extract quantitative information about the land surface from hyperspectral data. Radiometric calibration is an important process in the pre-processing of hyperspectral data. The radiometric calibration of airborne hyperspectral sensors is usually performed in the laboratory. However, the radiometric performance of these sensors can be reduced by the significant stresses generated during their transport, installation, and/or data acquisition [5]. Therefore, the radiometric calibration coefficients determined in the laboratory may not be appropriate for data acquired during the flight. Vicarious calibration methods are often used to produce a new set of radiometric calibration coefficients to replace those derived in the laboratory [6], [7]. For airborne hyperspectral sensors, a feasible vicarious calibration method is reflectance-based test site calibration [8], [9]. To perform a test site calibration for airborne hyperspectral sensors, portable or permanent reference reflectance targets must be deployed over the test sites. In addition, *in situ* measurements of target reflectance and atmospheric properties during the flight are required to predict the at-sensor radiances [10].

Besides radiometric calibration, quality assessment is also a key step in the pre-processing of hyperspectral data. The signal-to-noise ratio (SNR) is an important criterion for characterizing the quality of hyperspectral data. Accurate evaluation of the SNR is crucial to quantitatively analyze the data, and a high SNR is required to optimize the use of the data [11]. Therefore, bands with particularly low SNR must be discarded. Image-based SNR estimation is a feasible method to assess the quality of hyperspectral data [12]. Several methods have been developed to perform image-based SNR estimation [13], [14].

After the pre-processing of hyperspectral data, accurate removal of atmospheric absorption and scattering effects is required to extract land surface reflectance from remotely sensed data. The atmospheric absorption and scattering effects in remotely sensed data can be corrected by a number of physical-based methods [1]. In addition to atmospheric absorption and scattering effects, the adjacency effect must be considered during the retrieval of land surface reflectance from hyperspectral data. The magnitude of this effect directly depends on atmospheric turbidity and surface heterogeneity [15]. Therefore, the adjacency effect is the most intricate problem that must be solved when removing atmospheric effects from hyperspectral data [16].

To validate land surface reflectance derived from airborne hyperspectral data, *in situ* measurements must be collected. *In situ* measurements are used to evaluate the performance of the retrieval algorithms of land surface reflectance. The accuracies of the retrieval parameters are characterized by comparing the values of the retrieval parameters with the *in situ* measurements. The accuracies to which the retrieved values match the *in situ* measurements are used to further improve the performance of the retrieval algorithms of land surface reflectance. The objectives of this study are 1) to assess the radiometric performance of a new hyperspectral sensor onboard an unmanned aerial vehicle (UAV) and 2) to validate land surface reflectance retrieval from airborne hyperspectral data using *in situ* measurements.

### Test Site and Data

#### 1. Test site

To evaluate the in-flight performance of a new hyperspectral sensor onboard an UAV, a comprehensive field campaign was conducted over the Baotou test site (Inner Mongolia, China: 40.88°N, 109.53°E) on 3 September 2011. The Baotou test site is located in a rural area, is surrounded by agricultural parcels, and has an average ground elevation of approximately 1.3 km above sea level (ASL). The test site receives little precipitation and has a high percentage of cloud-free days. The area has a continental climate that is characterized by four seasons and a large diurnal temperature variation. The yearly average temperature is 6–7°C, and the average annual rainfall is 200–250 mm.

**Figure 1:**
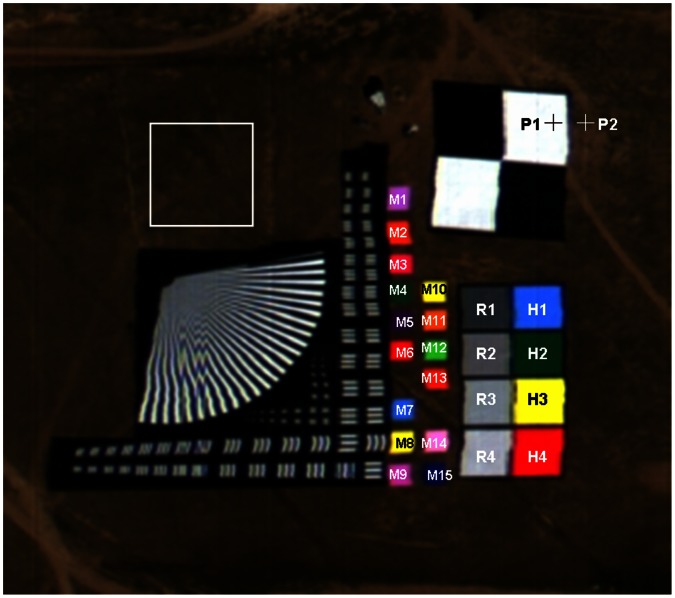
A subset image extracted from hyperspectral data acquired over the test site on 3 September 2011 at 06∶42 UTC. The locations of the 23 targets (R1–R4, H1–H4, and M1–M15) are displayed in the image. A bare area highlighted in a white rectangle is used to perform the signal-to-noise ratio estimation. The two pixels labeled as P_1_ and P_2_ are used to demonstrate the discrepancy between the uniform and non-uniform Lambertian land surface reflectance.

A number of portable reference reflectance targets were deployed over the test site. Figure 1 shows a subset image extracted from data acquired by the hyperspectral sensor onboard the UAV on 3 September 2011 at 06∶42 UTC. The targets denoted as R1–R4, H1–H4, and M1–M15 in Figure 1 are used in this study. Targets R1–R4 and H1–H4 are 15 m×15 m in size, while targets M1–M15 are 7 m×7 m in size. Targets R1–R4, which have nominal surface reflectance of 0.2, 0.3, 0.4, and 0.5, respectively, are used to perform the radiometric calibration of the hyperspectral sensor. Targets H1–H4 and M1–M15 are employed to evaluate the accuracies of the land surface reflectance retrieved from the hyperspectral data.

#### 2. UAV-HYPER sensor

**Figure 2:**
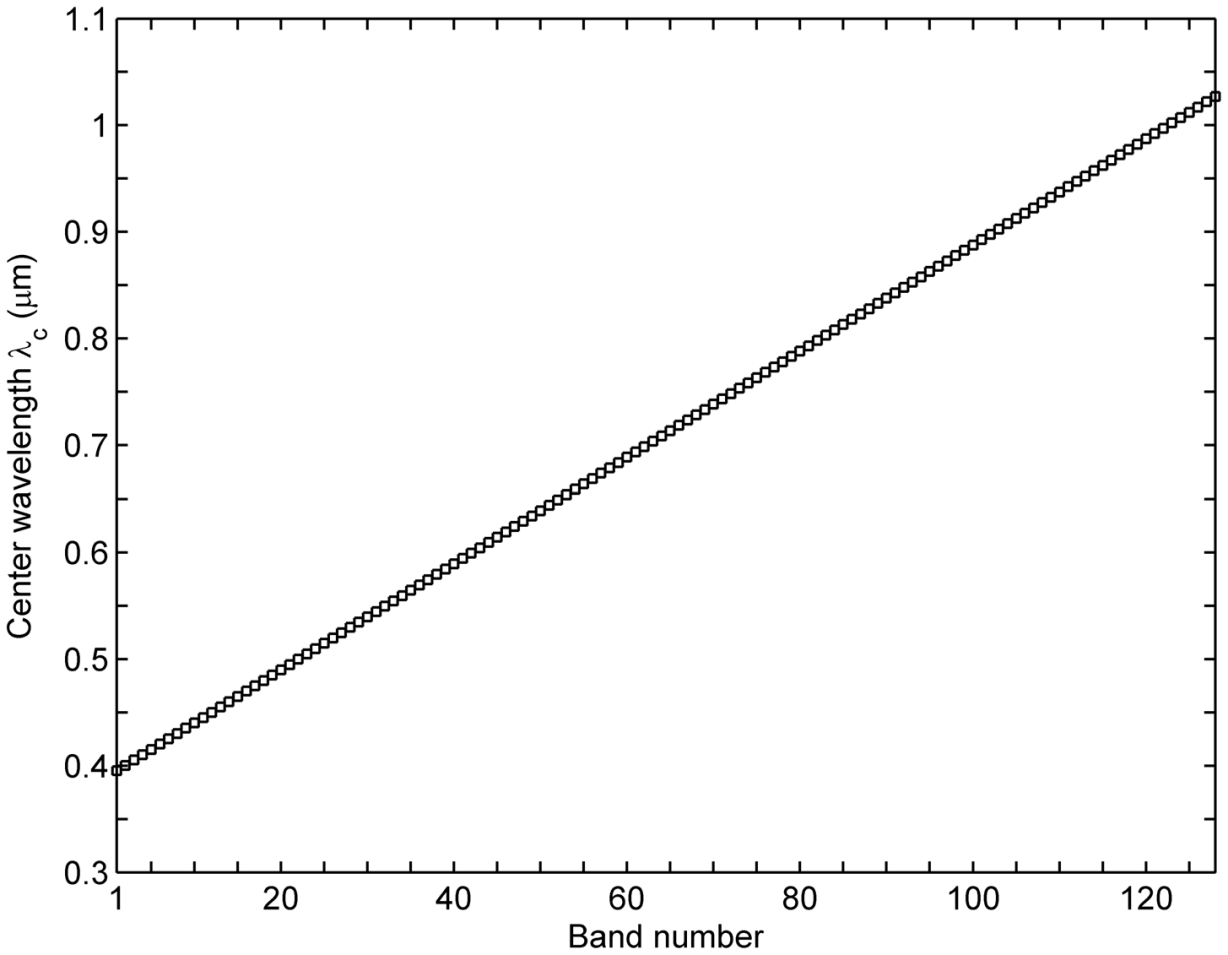
Center wavelengths 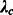 of 128 bands of the UAV-HYPER sensor.

The hyperspectral sensor, which was developed by the Changchun Institute of Optics, Fine Mechanics and Physics, Chinese Academy of Sciences, was installed on an UAV operated by the Research Institute of Unmanned Flight Vehicle Design, Beihang University, China. Hereafter, the hyperspectral sensor is referred to as UAV-HYPER. The UAV-HYPER sensor is a pushbroom scanner that utilizes linear CCD arrays. The main characteristics of the UAV-HYPER sensor are presented in Table 1. During the campaign, the operational altitude of the UAV-HYPER was approximately 3.5 km above ground level (AGL), which gives a spatial resolution of approximately 0.7 m at nadir. The UAV-HYPER image has an across-track sampling of 1024 pixels, which gives a swath width of approximately 0.7 km. The spectral response functions of the UAV-HYPER sensor are simulated using Gaussian functions with the center wavelengths and band widths that were measured during the laboratory calibration. The center wavelengths of 128 bands of the UAV-HYPER sensor are shown in Figure 2.

**Table 1:** Main characteristics of the UAV-HYPER sensor.

Parameter	Requirement
Instantaneous field of view	0.2 mrad
Field of view	11.5°
Pixel per line	1024
Spectral range	350–1030 nm
Spectral resolution	5 nm
Spatial resolution	1 m @ 5 km flight altitude AGL
Number of bands	128
Swath width	1 km @ 5 km flight altitude AGL
Digitization	12 bits
Signal-to-noise ratio	>100∶1

#### 3. In situ measurements

**Figure 3:**
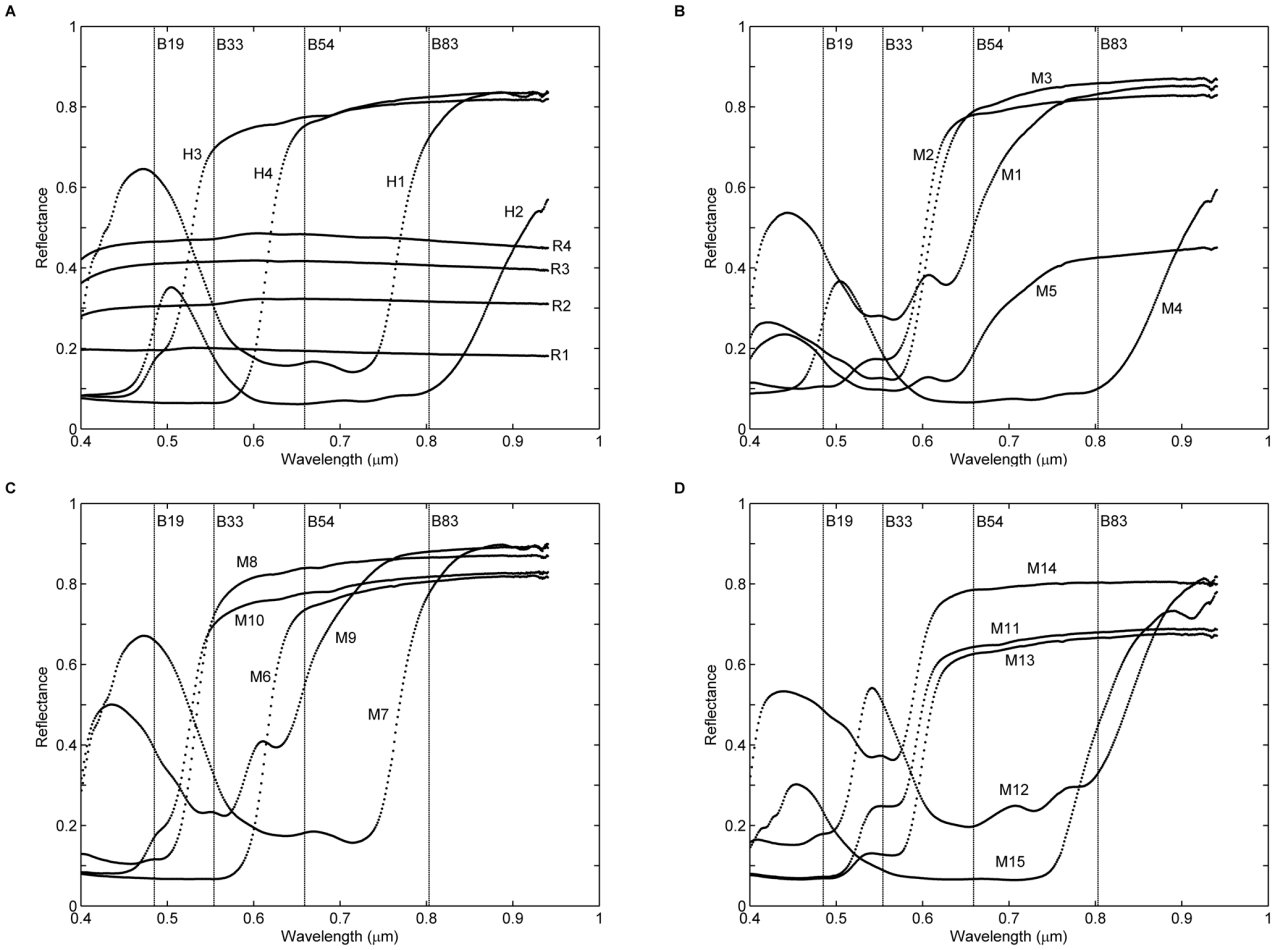
*In situ* surface reflectance spectra of the 23 targets (R1–R4, H1–H4, and M1–M15) in the wavelength range of 0.4–0.95 µm. The four dashed vertical lines denote the positions of the center wavelengths of four selected bands (19, 33, 54, and 83) that represent the blue, green, red, and near-infrared bands, respectively.

*In situ* measurements of the 23 targets (R1–R4, H1–H4, and M1–M15) were carried out to collect the surface reflectance spectra with a SVC HR-1024 field portable spectroradiometer at the time of the UAV-HYPER data acquisition. The spectroradiometer has 1024 channels that cover the spectral range from 350 to 2500 nm. A reference measurement was collected with a white Spectralon reference panel before and after each target measurement. The spectra were measured in absolute radiance mode at nadir. The raw spectra of each target were scaled with the reference measurements to produce reflectance spectra. Five measurements of each target were averaged to yield a representative reflectance spectrum. The averaged reflectance spectra of each of the 23 targets are shown in Figure 3. Because the wavelength range of the UAV-HYPER sensor is in the 0.4–1.03 µm region and the sensitivity of the Si detector of the SVC spectroradiometer is reduced around 1 µm, only the reflectance spectra in the wavelength range of 0.4–0.95 µm are plotted in Figure 3.

In addition to the surface reflectance measurements of the targets, aerosol optical depth (AOD) and columnar water vapor (CWV) were also collected with an automatic CIMEL CE318 sunphotometer. The sunphotometer has nine channels at nominal wavelengths of 340, 380, 440, 500, 670, 870, 936, 1020, and 1640 nm. Measurements at 936 nm were used to derive the CWV [17] with the coefficients simulated by MODTRAN [18]. The AOD at 550 nm was derived from the other channels using the Ångström law. Detailed information on the method used to retrieve the AOD can be found in [19]. The measured values of the AOD at 550 nm (AOD@550) and the CWV at the time of the UAV-HYPER data acquisition are 0.18 and 1.7 g cm^−2^, respectively.

### Radiometric Performance of the UAV-HYPER Sensor

#### 1. SNR estimation

Some bands of the UAV-HYPER data have low SNR values. A method based on local means and local standard deviations of small imaging blocks is used to estimate the SNR from the UAV-HYPER data. A 3×3 pixel window is chosen as the block size, and the SNR is calculated as the ratio of the average signal to the average noise of the UAV-HYPER data. The detailed procedure to estimate the SNR from data acquired with imaging spectrometers can be found in [14].

**Figure 4:**
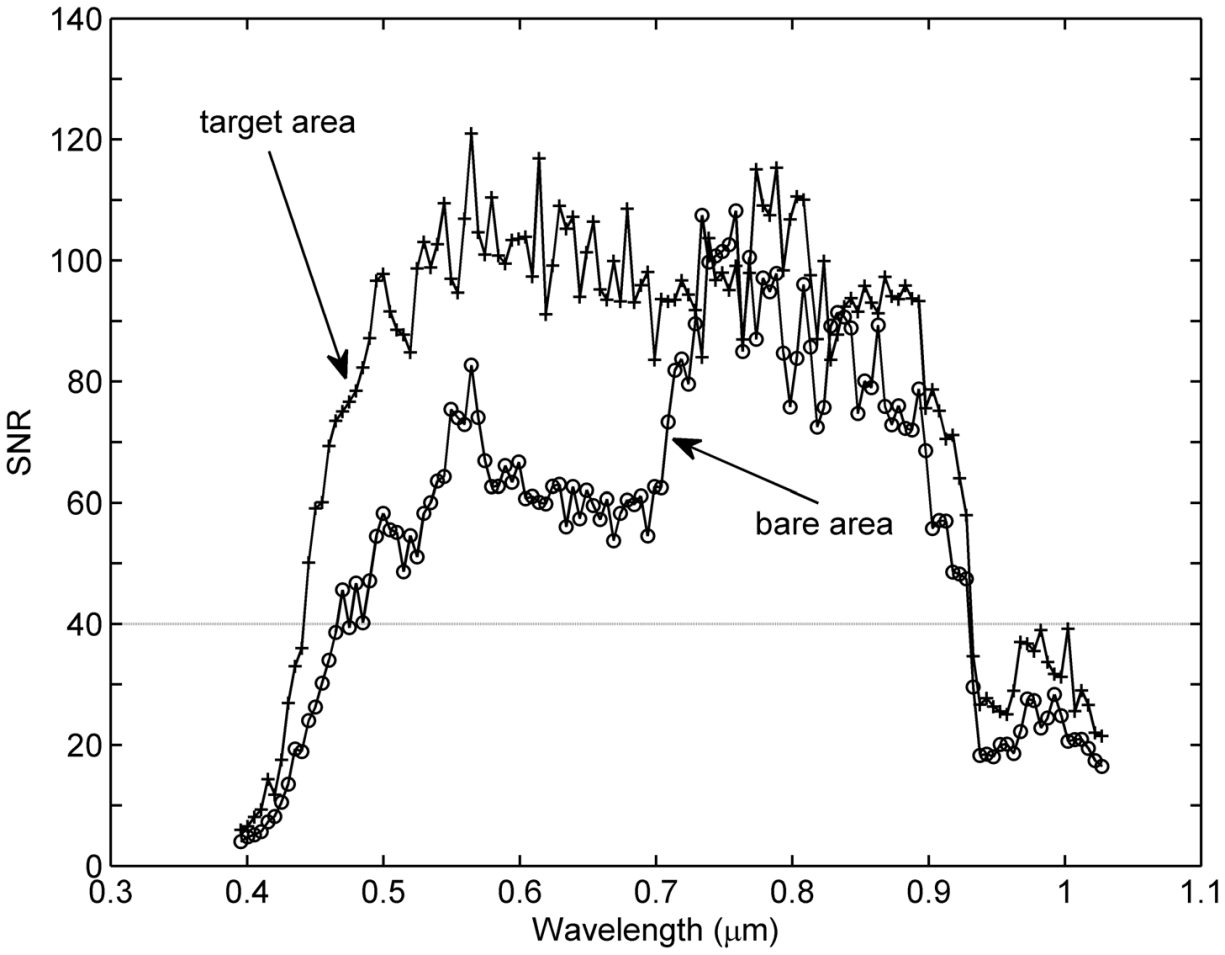
Signal-to-noise ratio estimated using the bare area shown in Figure 1 and the target area including targets R1–R4 and H1–H4. The bands with signal-to-noise ratio values lower than 40 are discarded in this study.

Figure 4 shows the SNR estimated using a bare area (50×50 pixels) shown in Figure 1. The SNR values are in the range of 4–110. Low SNR can be found in the first and last bands of the UAV-HYPER sensor. For comparison, the SNR is also estimated using targets R1–R4 and H1–H4. An area of 10×10 pixels is extracted from the center of each of the eight targets to estimate the SNR. The SNR values of the eight targets are then averaged to yield a single averaged SNR. The results are also shown in Figure 4. The SNR estimated using the target area range from approximately 5 to 120. Low SNR values can also be found in the first and last bands of the UAV-HYPER data. Except for the bands with low SNR, the SNR values estimated using the target area are slightly greater than those estimated using the bare area. This is because the target area is more homogeneous than the bare area. Therefore, the SNR of the target area is more suitable to characterize the quality of the UAV-HYPER data. To minimize the effect of low SNR, the bands with SNR values lower than 40 are discarded. Therefore, only bands 13–108, with SNR values between 40 and 120, are used in the following analysis.

#### 2. Radiometric calibration of the UAV-HYPER sensor

Radiometric calibration coefficients generally differ from the laboratory pre-flight values due to in-flight changes in instrument behavior, such as optical defocusing or distortion of the dispersed radiation on the detector arrays. The procedure that converts the digital number (DN) to the at-sensor radiance *L_sensor_* according to the radiometric calibration coefficients is called radiometric calibration and can be given by:
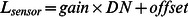
(1)where *gain* and *offset* are the radiometric calibration coefficients.

To determine the radiometric calibration coefficients, the at-sensor radiance *L_sensor_* is calculated using Equation (2) assuming a uniform Lambertian surface:
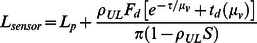
(2)where *L_p_* is the atmospheric path radiance, 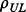 is the uniform Lambertian land surface reflectance, *F_d_* is the total solar flux at ground level, 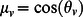 is the cosine of the view zenith angle 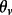, 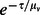 and 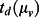 are the direct and diffuse transmittances in the viewing direction, and *S* is the spherical albedo of the atmosphere.

In this study, the apparent reflectance corresponding to the at-sensor radiance *L_sensor_* calculated using Equation (1) is denoted as 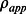, while the at-sensor reflectance corresponding to the at-sensor radiance *L_sensor_* simulated using Equation (2) is referred to as 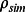.

**Figure 5:**
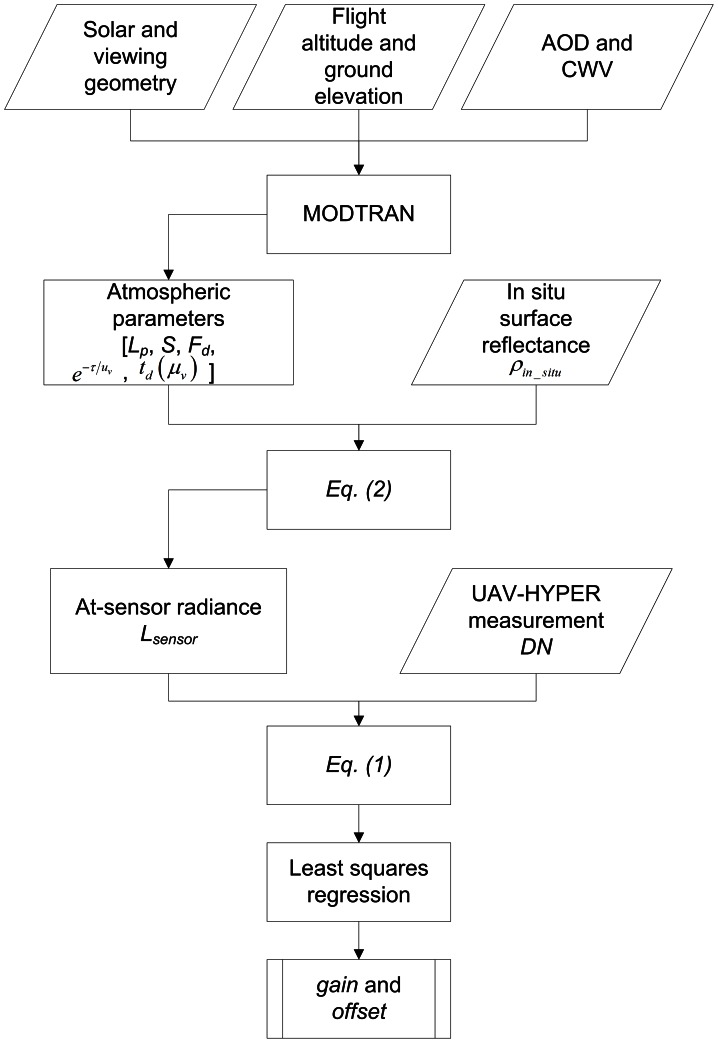
Flowchart of the radiometric calibration procedure of the UAV-HYPER sensor.

Targets R1–R4 are used to determine the radiometric calibration coefficients of the UAV-HYPER sensor. The DNs are averaged over a 3×3 pixel window, which is extracted from the center of each of the four targets. The at-sensor radiances *L_sensor_* are calculated using Equation (2) in conjunction with five atmospheric parameters (*L_p_*, *S*, *F_d_*, 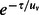, and 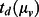) simulated by MODTRAN and the measured surface reflectance shown in Figure 3. The input parameters to MODTRAN for the radiative transfer calculations are a mid-latitude summer atmosphere, rural aerosol with AOD@550 of 0.18, CWV of 1.7 g cm^−2^, flight altitude (FA) of 4.8 km ASL, ground elevation (GE) of 1.3 km ASL, solar zenith angle (SZA) of 44.1°, viewing zenith angle (VZA) of 2.5°, and relative azimuth angle (RAA) of 42.5°/137.5°. The radiometric calibration coefficients (*gain* and *offset*) are obtained by a least squares regression from Equation (1) using the DNs and the corresponding at-sensor radiances *L_sensor_* of the four targets (R1–R4). Figure 5 shows a flowchart of the radiometric calibration procedure of the UAV-HYPER sensor.

**Figure 6:**
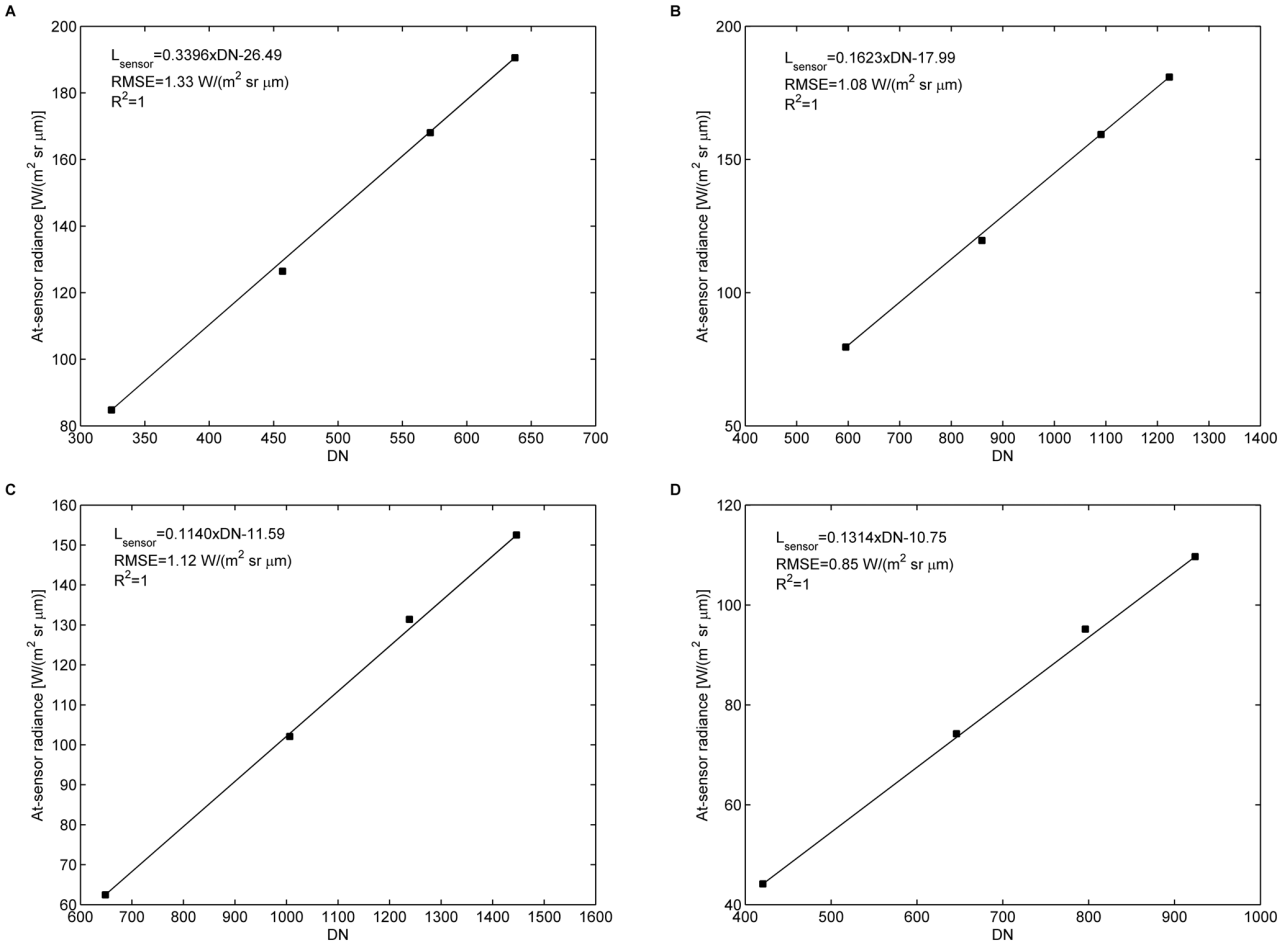
At-sensor radiances *L_sensor_* as a function of the DN for bands 19, 33, 54, and 83. 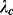
 is the center wavelength.

Four bands (band 19 centered at 0.485 µm, band 33 at 0.554 µm, band 54 at 0.659 µm, and band 83 at 0.803 µm) are arbitrarily selected to represent the blue, green, red, and near-infrared bands, respectively. The positions of the center wavelengths of the four bands are shown in Figure 3. The at-sensor radiances *L_sensor_* as a function of the DNs for the four bands are shown in Figure 6. The linear response of the UAV-HYPER sensor is good for the four bands, with R^2^ of approximately 1 and root mean square error (RMSE) of approximately 1 W/(m^2^ sr µm). The other bands have similar performance, which is not shown.

**Figure 7:**
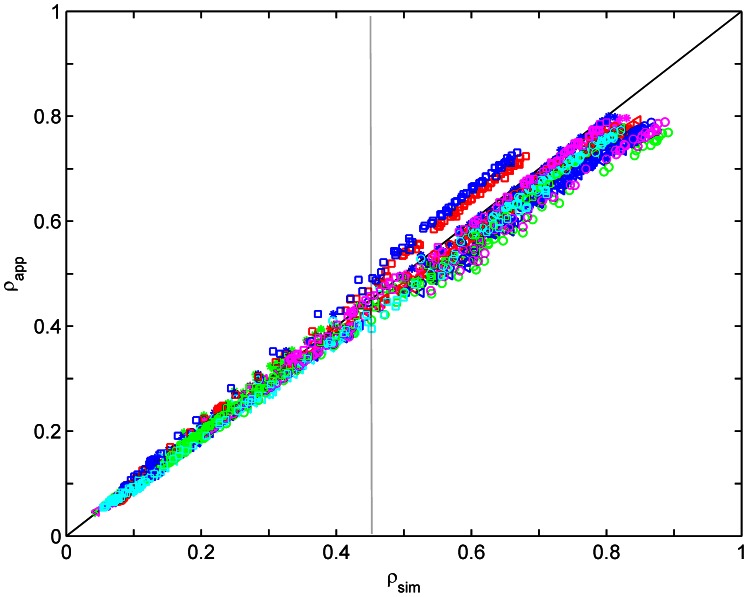
Comparison of the apparent reflectance 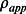 with the simulated at-sensor reflectance 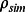 for targets H1–H4 and M1–M15 in bands 13–108. Different symbols with different colors represent different targets.

To further demonstrate the linear response range of the UAV-HYPER sensor, Figure 7 shows the apparent reflectance 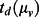 versus the simulated at-sensor reflectance 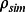 for the 19 targets (H1–H4 and M1–M15) in bands 13–108. Different symbols with different colors represent different targets. As shown in Figure 7, 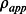 matches 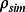 well in the apparent reflectance range of approximately 0.05–0.45. The result illustrates that the linear response of the UAV-HYPER sensor is good in this apparent reflectance range. Nevertheless, 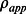 does not correspond well to 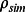 when 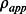 is greater than approximately 0.45. This may be due to the non-linear response of the UAV-HYPER sensor beyond 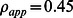 and/or errors in the *in situ* measurements.

To evaluate the accuracies of the radiometric calibration of the UAV-HYPER sensor, the RMSE and relative RMSE (RRMSE) between 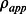 and 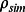 for targets H1–H4 and M1–M15 in bands 13–108 are calculated according to Equations (3) and (4):
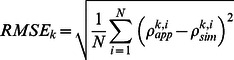
(3)

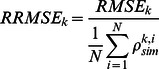
(4)where *k* is the band number, *i* is the target number, and *N* = 19 (targets H1–H4 and M1–M15).

**Figure 8:**
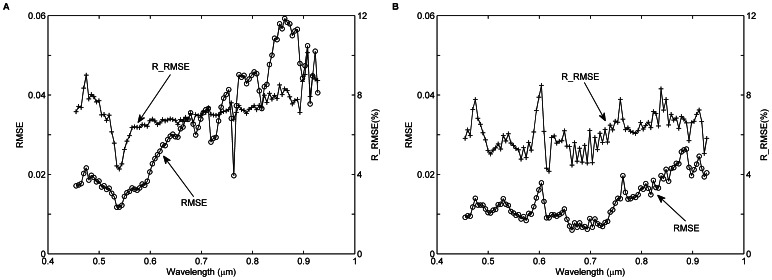
Root mean square error (RMSE) and relative RMSE (RRMSE) values of apparent reflectance as a function of the wavelength for targets H1–H4 and M1–M15. A: RMSE and RRMSE values between the apparent reflectance 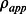 and the simulated at-sensor reflectance 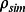 for targets H1–H4 and M1–M15 in bands 13–108. B: Same as Figure 8A, but the apparent reflectance greater than 0.45 in bands 13–108 has been discarded due to the non-linear response of the UAV-HYPER sensor.

Figure 8A displays the RMSE and RRMSE values between 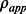 and 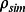 calculated from targets H1–H4 and M1–M15 in bands 13–108. The RMSE values are between approximately 0.01 and 0.06. The larger RMSE values occur in the near-infrared region, where most of the apparent reflectance is beyond the linear response range of the UAV-HYPER sensor. Conversely, the smaller RMSE values occur in the visible range, where most of the apparent reflectance is in the linear response range of the UAV-HYPER sensor. The RRMSE values are between approximately 4% and 10%. The larger RRMSE values are approximately 10% and occur in the first and last bands of the UAV-HYPER sensor. The smaller RRMSE values are approximately 4% and are approximately 0.54 µm.

To further examine the RMSE and RRMSE values between 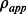 and 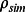 in the linear response range of the UAV-HYPER sensor, apparent reflectance greater than 0.45 in bands 13–108 were discarded. The results are shown in Figure 8B. The range of RMSE values is approximately 0.005 to 0.03, which is less than those in Figure 8A. The RRMSE values are between approximately 3% and 9%, which are slightly less than those in Figure 8A.

### Methodology

#### 1. Retrieval of uniform lambertian land surface reflectance

The compilation of a large atmospheric look-up table (LUT) is useful in deriving land surface reflectance from airborne hyperspectral data, especially for the operational atmospheric processing of large volumes of data [20]. MODTRAN is used to establish the atmospheric LUT because of its high accuracy and fine spectral resolution. A mid-latitude summer atmospheric model is selected. Multiple scattering is calculated using the scaled DIScrete Ordinate Radiative Transfer (DISORT) option of MODTRAN with eight streams. The carbon dioxide (CO_2_) mixing ratio of the atmosphere is set to 380 parts per million by volume (ppmv). Due to its low spatial and temporal variations, the total ozone column content is fixed at 0.33 atm-cm for ground at sea level. The rural aerosol model is selected to represent aerosol in areas that are not strongly affected by urban or industrial sources. The radiative transfer calculations are performed using the default MODTRAN 5 cm^−1^ atmospheric database.

Six free parameters are selected as inputs in the atmospheric LUT: AOD@550, CWV, FA, GE, SZA, and RAA. Due to the small FOV of the UAV-HYPER sensor (FOV = 11.5°), VZA is fixed at 2.5° for the establishment of the atmospheric LUT. An AOD@550 range of 0.05–1.5 is used to characterize clean to very turbid atmospheric conditions. A CWV range of 0.1–5 g cm^−2^ represents a normal range for a mid-latitude summer atmosphere. The maximum flight altitude of the UAV is 7 km. The ground elevation at the Baotou test site is between 0 and 2.5 km. SZA ranges from 0° to 70° with an increment of 10°, and RAA ranges from 0° to 180° with an increment of 30°. The breakpoint positions in the atmospheric LUT for the six input parameters are presented in Table 2. The number of breakpoints describing each dimension in the atmospheric LUT is selected as a trade-off between sufficient sampling and LUT size. Given a certain set of inputs, the values of the atmospheric parameters are calculated through linear interpolation in the six directions of the parameter space [21]. The atmospheric LUT gives five atmospheric parameters as outputs: *L_p_*, *S*, *F_d_*, 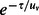, and 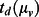. Detailed information on calculating the atmospheric parameters can be found in [22].

**Table 2:** Breakpoint positions in the atmospheric LUT for the six input parameters.

Parameter*	#1	#2	#3	#4	#5	#6	#7	#8
AOD@550	0.05	0.1	0.3	0.6	1.0	1.5	―	―
CWV (g cm^−2^)	0.1	0.5	1.5	2.5	3.5	5.0	―	―
FA (km)	1	2	3	4	5	6	7	―
GE (km)	0	0.5	1.0	1.5	2.0	2.5	―	―
SZA (°)	0	10	20	30	40	50	60	70
RAA (°)	0	30	60	90	120	150	180	―

* AOD@550: aerosol optical depth at 550 nm, CWV: columnar water vapor, FA: flight altitude, GE: ground elevation, SZA: solar zenith angle, and RAA: relative azimuth angle.

Once the atmospheric parameters are determined, 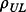 can be calculated from *L_sensor_* by inverting Equation (2) on a pixel-by-pixel basis:
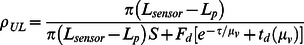
(5)


**Figure 9:**
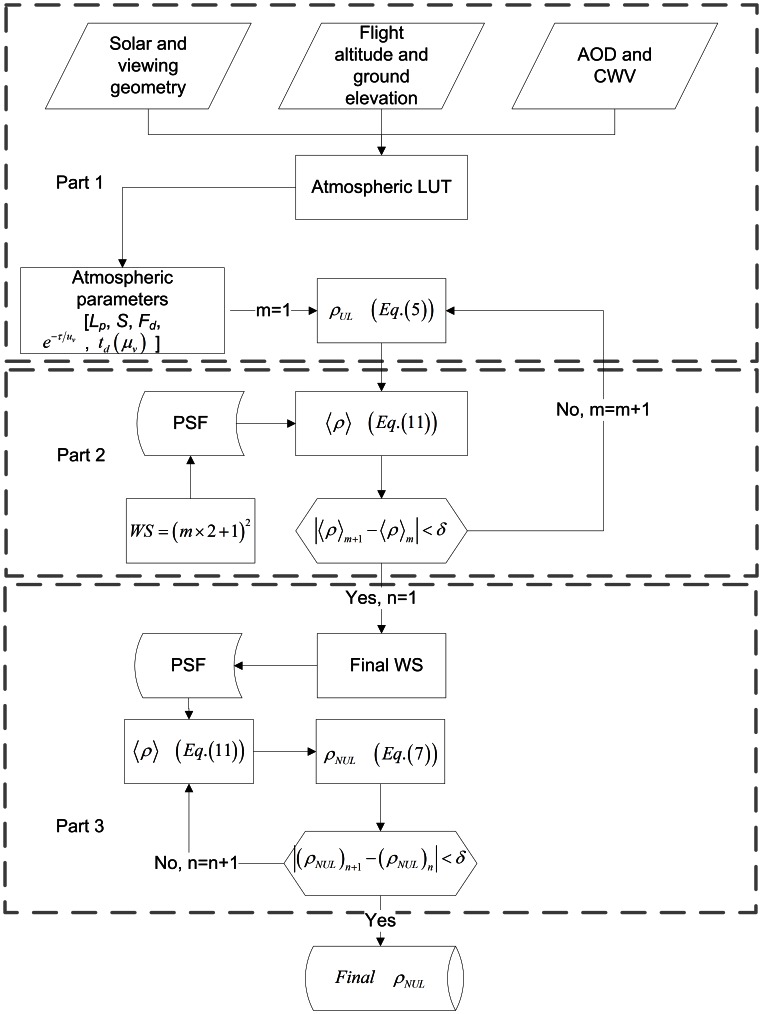
Flowchart of the atmospheric correction procedure for the UAV-HYPER data. Part 1 is used to derive the uniform Lambertian surface reflectance 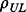 using the atmospheric look-up table (LUT). Part 2 is used to determine the window size (WS) of the atmospheric point spread function (PSF) by calculating the 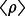 difference between two successive iterations. Part 3 is used to determine the final non-uniform Lambertian surface reflectance 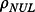 by calculating the 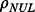 difference between two successive iterations.

The procedure for retrieving 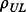 is shown in Part 1 of Figure 9.

#### 2. Retrieval of non-uniform lambertian land surface reflectance

For the case of a non-uniform Lambertian surface, Equation (2) can be rewritten as:
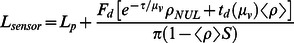
(6)


The non-uniform Lambertian land surface reflectance 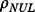 can then be calculated from *L_sensor_* by inverting Equation (6) on a pixel-by-pixel basis:
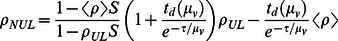
(7)where the average reflectance of the surrounding 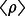 can be weighted by an atmospheric point spread function (PSF) that takes into account the contribution of the surrounding region according to the distance from the target [23]:



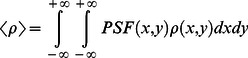
(8)According to [24], the unnormalized atmospheric PSF is defined as:
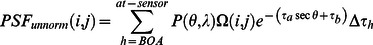
(9)where *h* is the atmospheric layer height, BOA is the bottom of atmosphere, 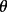 is the scattering angle, 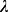 is the wavelength, 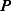 is the scattering phase function, 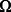 is the solid angle subtended by the unit cross section as seen by the (*i*, *j*)th surrounding pixel, 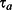 is the atmospheric optical depth from the surrounding pixel to the atmospheric layer height *h*, 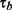 is the atmospheric optical depth from the atmospheric layer height *h* to the sensor, and 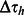 is the atmospheric optical depth at the atmospheric layer height *h*.

To calculate the weight of each surrounding pixel, it is necessary to normalize the atmospheric PSF (i.e. the atmospheric PSF must integrate to unity). Assuming that the atmosphere is homogeneous within each atmospheric layer, 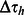 can be canceled, leaving [24]:
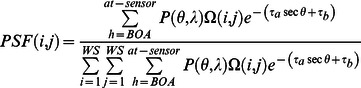
(10)where WS is the moving window size, which depends on the pixel size, the atmospheric parameters, the spectral band, and the spatial frequencies of the image itself [25].

The average reflectance of the surrounding 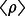 can then be calculated in the discrete form of Equation (8), namely:
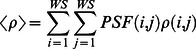
(11)


To determine the window size WS, an iterative method is used to calculate the 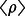 difference between two successive iterations ‘*m*’ and ‘*m+*1’. If the difference is less than 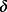 (e.g. the noise equivalent reflectance of the sensor at that wavelength), the iterative process is stopped. Otherwise, the iteration procedure goes back to recalculate 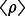, and the order ‘*m*’ is increased by 1. The iteration procedure is shown in the Part 2 of Figure 9. The outputs of the iteration procedure are the final WS image and the initial 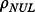 image.

In theory, the reflectance 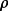 on the right-hand side of Equation (11) should be the actual reflectance; however, the actual reflectance is not available at this stage. Therefore, an iteration procedure is used to reduce the error introduced by replacing the actual reflectance with 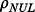 [26]. The iteration procedure is shown in Part 3 of Figure 9. The output of the iteration procedure is the final 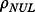 image.

## Results and Discussion

### 1. Results of Uniform Lambertian Land Surface Reflectance Retrieval

**Figure 10:**
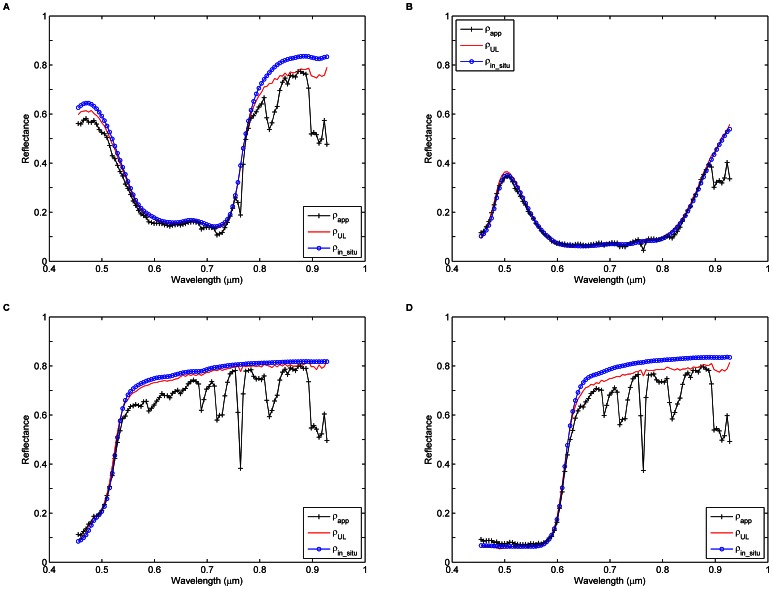
Comparison of the uniform Lambertian surface reflectance 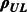 derived using Equation (5) with the apparent reflectance 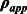 and the *in situ* surface reflectance 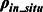 for targets H1–H4 in bands 13–108.

The *in situ* land surface reflectance measurements of the 19 targets (H1–H4 and M1–M15) are used to evaluate the accuracies of the atmospheric correction of the UAV-HYPER data. A 3×3 pixel window is selected from the center of each of the 19 targets to yield the average surface reflectance. The uniform Lambertian land surface reflectance 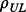 derived from Equation (5) is compared with the apparent reflectance 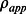 and the *in situ* surface reflectance 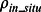 for targets H1–H4 in bands 13–108 in Figure 10. The absorption effects of oxygen (0.76 µm) and water vapor (0.82 µm) are clearly observed in 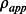 but have been nearly removed in 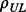. These results demonstrate that the spectral shift of the UAV-HYPER sensor is small around the oxygen absorption feature centered at 0.76 µm. However, small dips can still be found at approximately 0.76 µm in targets H3 and H4; this is most likely because the oxygen concentration given in the radiative transfer calculations is lower than the actual conditions. 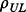 generally agrees closely with 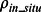 for targets H1–H4. However, large discrepancies are present in some bands; these may be caused by radiometric calibration errors of the UAV-HYPER sensor and/or the radiative transfer calculations.

**Figure 11:**
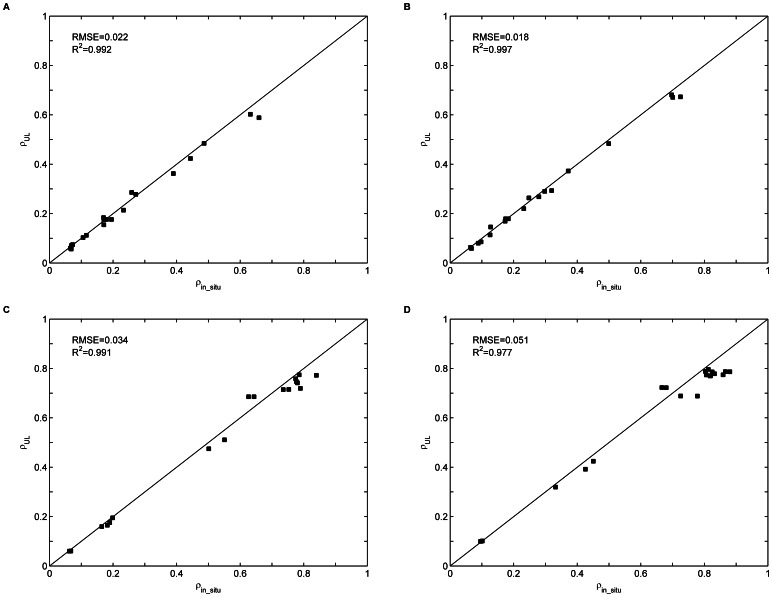
Comparison of the uniform Lambertian surface reflectance 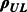 derived using Equation (5) with the *in situ* surface reflectance 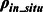 for targets H1–H4 and M1–M15 in bands 19, 33, 54, and 83. 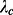
 is the center wavelength of each of the four bands.

Figure 11 shows 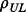 versus 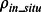 for targets H1–H4 and M1–M15 in bands 19, 33, 54, and 83. The results show that 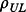 generally agrees well with 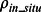 in these four bands, with R^2^ values of 0.992, 0.997, 0.991, and 0.977 and RMSE values of 0.022, 0.018, 0.034, and 0.051, respectively. However, they do not match well in the high reflectance conditions, as is shown in Figures 11C and D.

**Figure 12:**
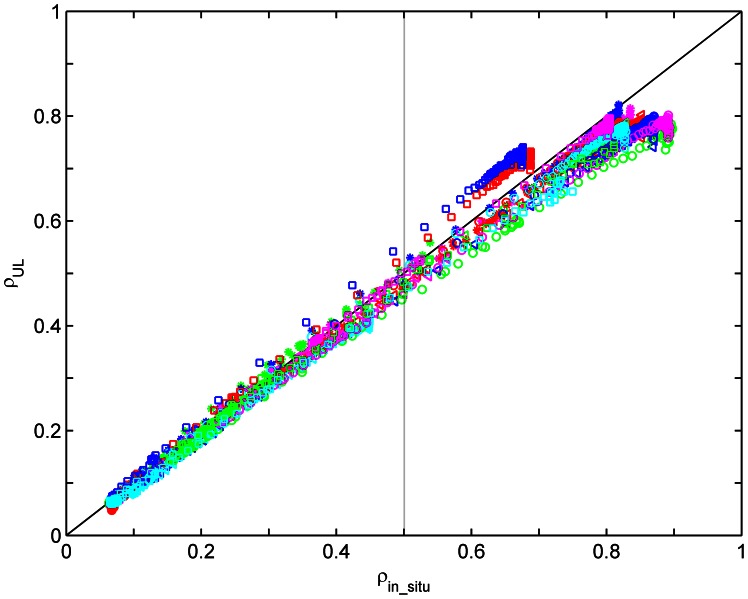
Comparison of the uniform Lambertian surface reflectance 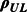 derived from Equation (5) and the *in situ* surface reflectance 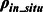 for targets H1–H4 and M1–M15 in bands 13–108. Different symbols with different colors represent different targets.

To further analyze these results, Figure 12 shows 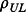 versus 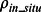 for targets H1–H4 and M1–M15 in bands 13–108. Different symbols with different colors represent different targets. The results show that 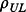 does not match 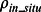 well when 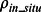 is greater than approximately 0.5. This discrepancy is believed to be mainly caused by the large errors of the radiometric calibration of the UAV-HYPER sensor due to its non-linear response when the surface reflectance is greater than approximately 0.5 and/or by measurement errors of 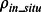. Nevertheless, 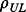 matches 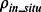 well in the surface reflectance range between approximately 0.05 and 0.5, which demonstrates the good accuracy of land surface reflectance retrieval from the UAV-HYPER data in the linear response range of the UAV-HYPER sensor.

### 2. Results of Non-uniform Lambertian Land Surface Reflectance Retrieval

**Figure 13:**
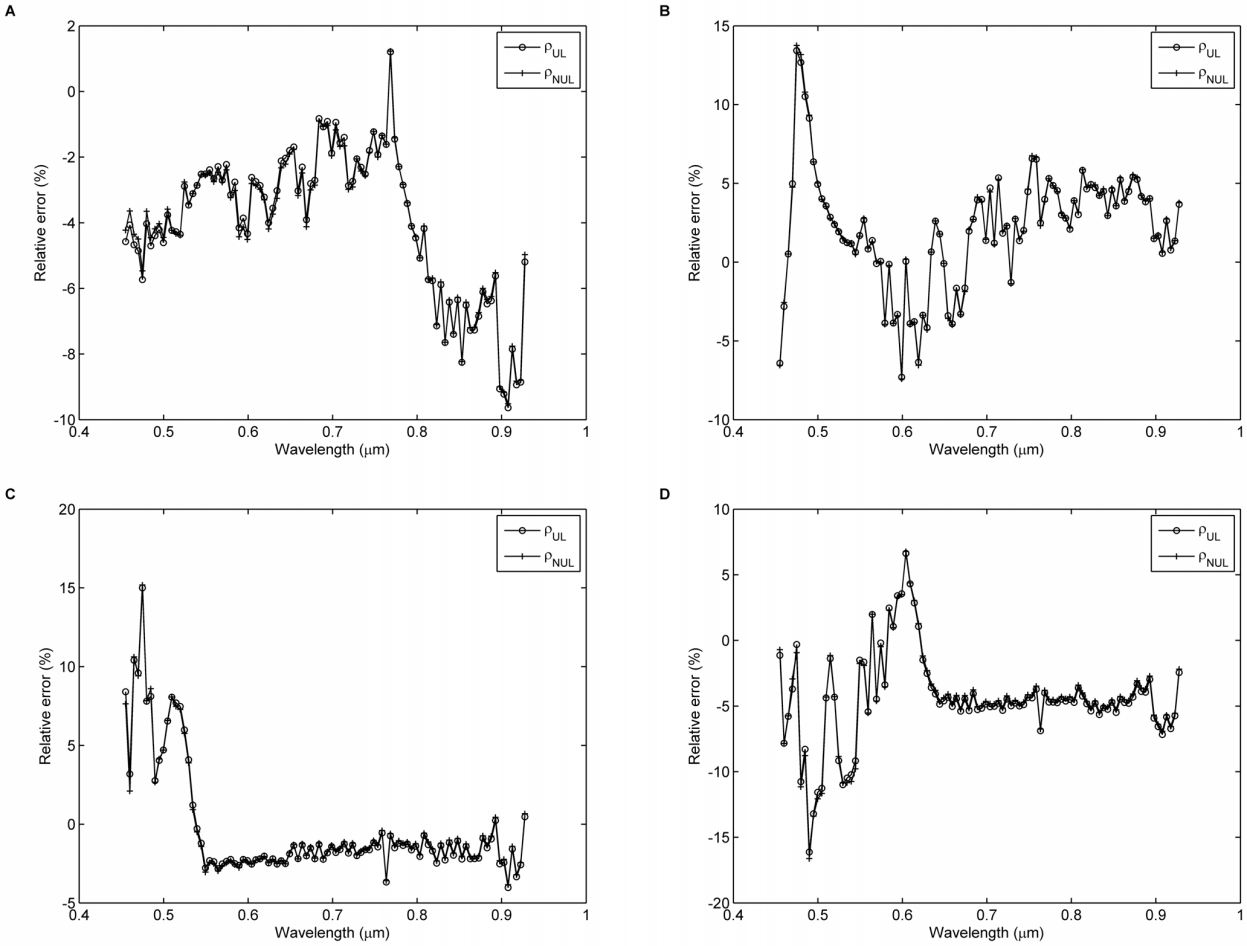
Relative errors of surface reflectance as a function of the wavelength for targets H1–H4. Relative errors between the uniform Lambertian surface reflectance 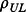 derived from Equation (5) and the non-uniform Lambertian surface reflectance 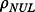 derived from Equation (7) and the *in situ* surface reflectance 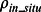 for targets H1–H4 in bands 13–108.

Figure 13 shows the relative errors between 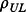 as well as the non-uniform Lambertian land surface reflectance 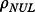 derived from Equation (7) and 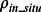 for targets H1–H4 in bands 13–108. The relative errors are less than 10% in most bands. Compared with Figure 10, large relative errors occur in the bands with wavelengths less than approximately 0.5 µm and surface reflectance less than approximately 0.4. This occurs because a small absolute difference for a low surface reflectance may lead to a large relative error. There is no evident improvement and difference in terms of the relative errors between 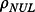 and 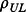 for targets H1–H4. Two reasons can explain these findings. One reason is that the AOD was relatively low at the time of the UAV-HYPER data acquisition (AOD = 0.18). The other reason is that the targets are large and have relatively homogeneous surface reflectance. Both these effects lead to a small difference between 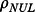 and 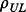.

**Figure 14:**
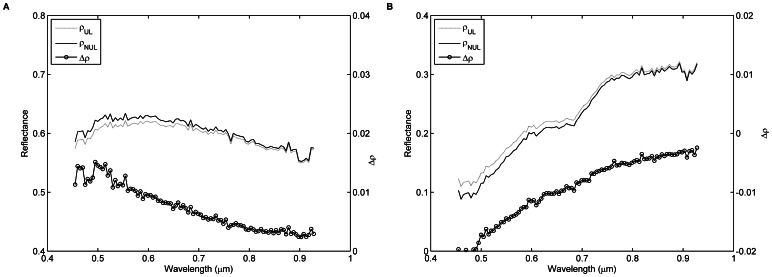
Comparison of the uniform Lambertian surface reflectance 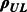 derived from Equation (5) and the non-uniform Lambertian surface reflectance 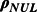 derived from Equation (7) for pixels (A) P_1_ and (B) P_2_. 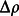
 is the surface reflectance difference between 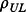 and 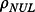 (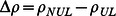).

Two pixels, labeled as P_1_ and P_2_ in Figure 1, are selected to calculate the discrepancy between 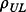 and 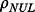. As shown in Figure 14, the surface reflectance of pixel P_1_ (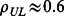) is greater than that of pixel P_2_ (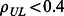 for all bands). Therefore, 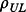 of pixel P_1_ is lower than its actual surface reflectance because photons escaping from the FOV of the UAV-HYPER sensor are not counterbalanced by those coming from the surrounding pixels (e.g. pixel P_2_). In contrast, 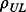 of pixel P_2_ is greater than its actual surface reflectance because more photons come from the surrounding pixels (e.g. pixel P_1_) than escape from the FOV of the UAV-HYPER sensor. As shown in Figure 14, because the surface reflectance of pixel P_1_ is greater than that of its surrounding pixels, 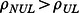 for the pixel P_1_. Furthermore, the surface reflectance difference 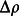 (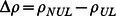) for pixel P_1_ decreases as wavelength increases because the effect of atmospheric scattering in the near-infrared region is less than in the visible region, where scattering from atmospheric aerosols dominates. In contrast, 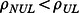, and absolute 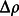 decreases as wavelength increases for pixel P_2_.

To further demonstrate the impact of AOD on the discrepancy between 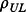 and 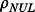, four AOD@550 values (0.05, 0.3, 0.5, and 1.0) are used to calculate the surface reflectance differences 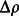 for pixels P_1_ and P_2_. The AOD@550 values of 0.05, 0.3, 0.5, and 1.0 represent clear, slightly turbid, turbid, and very turbid atmospheric conditions, respectively. To simulate the at-sensor radiances of the UAV-HYPER sensor, the other input parameters for the radiative transfer calculations are the same as those used to perform the atmospheric correction of the UAV-HYPER data. Furthermore, the image of 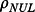 is used as a reference image of surface reflectance. The average reflectance 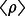 of each pixel is simulated by Equation (11) using the atmospheric PSF and the reference image of surface reflectance, and the at-sensor radiances for the four AOD@550 values are simulated using Equation (6). The images of 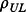 and 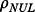 for the four AOD@550 values are then derived from the corresponding at-sensor radiances using Equations (5) and (7), respectively.

**Figure 15:**
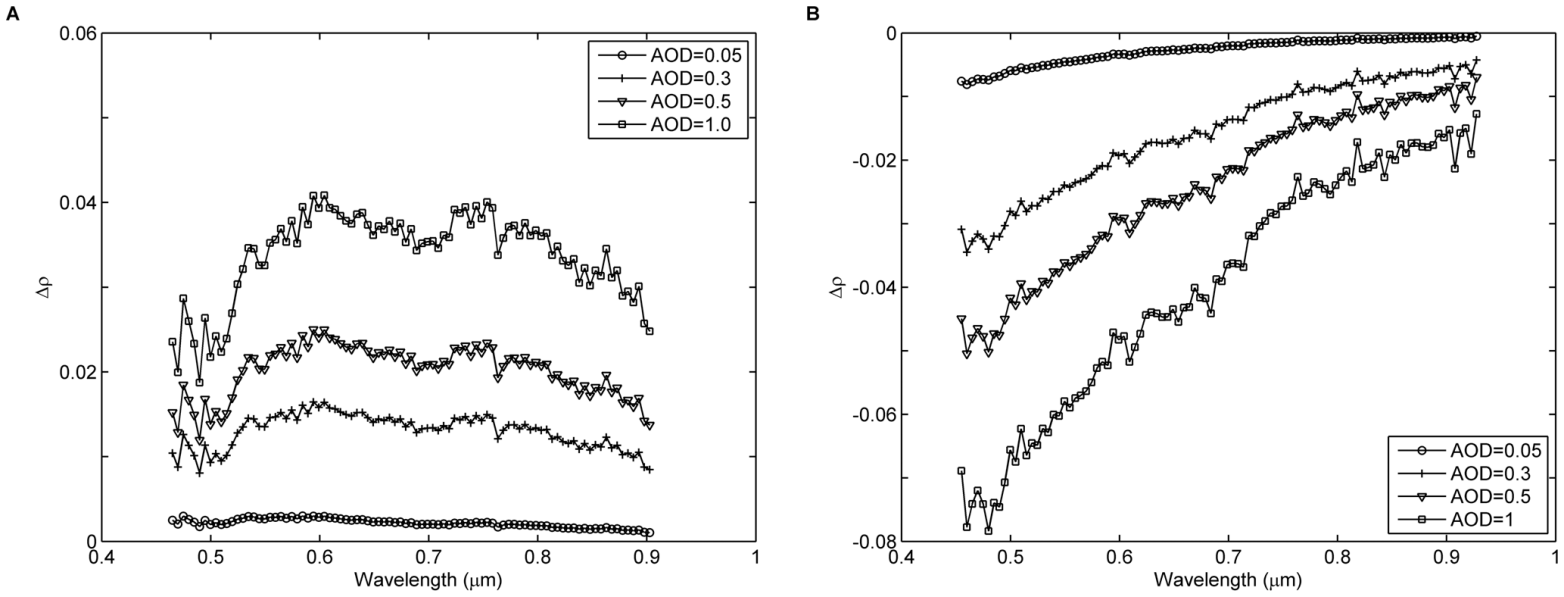
Surface reflectance differences 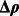 (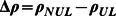) as a function of the wavelength for the four AOD@550 values for pixels (A) P_1_ and (B) P_2_.

The surface reflectance differences 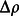 for the four AOD@550 values are shown in Figure 15. For pixel P_1_, 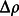 decreases as wavelength increases for each AOD@550 value. In addition, 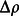 increases as the AOD@550 value increases for each band. The maximum 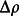 for the case of AOD@550 = 1.0 can reach 0.035 in the blue region and is approximately seven times larger than that for the case of AOD@550 = 0.05. The relative surface reflectance difference 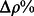 (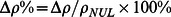) varies from approximately 1% in the blue region to approximately 0% in the near-infrared region for the case of AOD@550 = 0.05, while 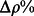 varies from approximately 6% in the blue region to approximately 2% in the near-infrared region for the case of AOD@550 = 1.0. For pixel P_2_, 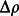 is negative, and its absolute value 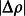 decreases as wavelength increases for each AOD@550 value. In addition, 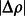 increases as the AOD@550 value increases for each band. The maximum 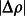 for the case of AOD@550 = 1.0 reaches 0.08 in the blue region and is approximately eight times larger than that for the case of AOD@550 = 0.05. The relative surface reflectance difference 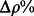 varies from approximately −10% in the blue region to approximately 0% in the near-infrared region for the case of AOD@550 = 0.05, while 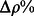 varies from approximately −80% in the blue region to approximately −7% in the near-infrared region for the case of AOD@550 = 1.0.

**Figure 16:**
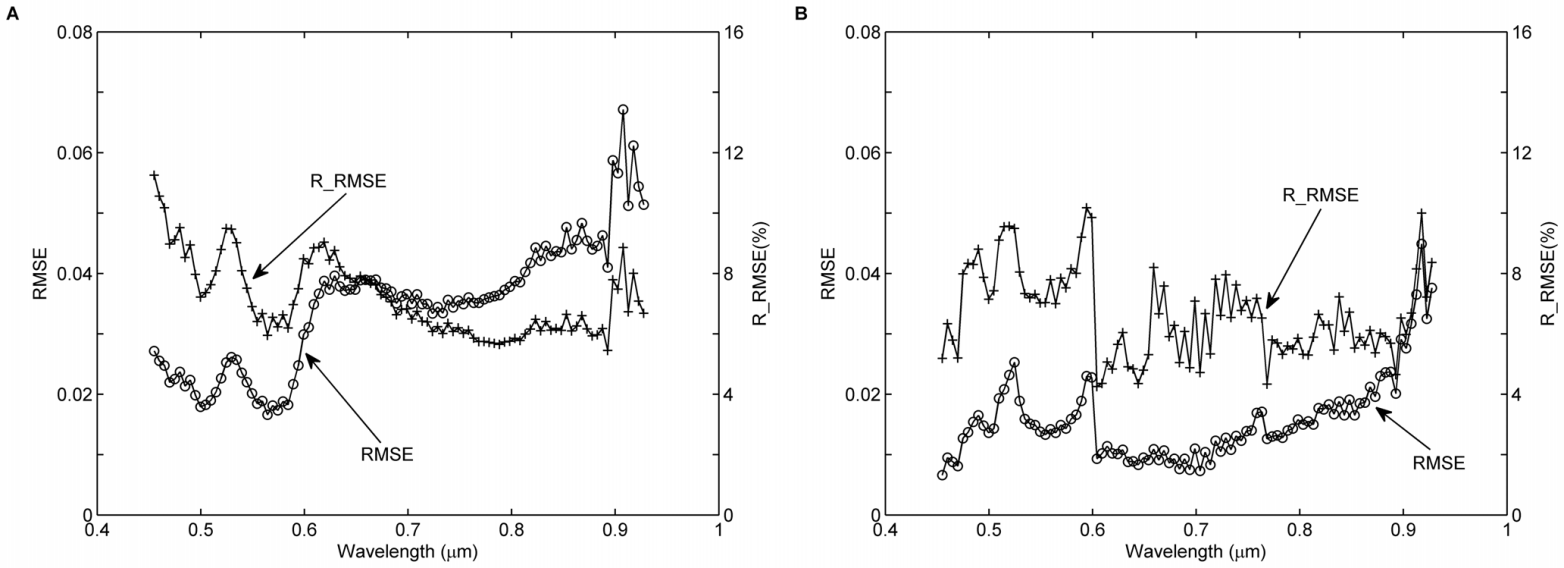
Root mean square error (RMSE) and relative RMSE (RRMSE) values of surface reflectance as a function of the wavelength for targets H1–H4 and M1–M15. A: RMSE and RRMSE values between the non-uniform Lambertian surface reflectance 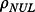 derived using Equation (7) and the *in situ* surface reflectance 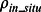 for targets H1–H4 and M1–M15 in bands 13–108. B: Same as Figure 16A, but surface reflectance greater than 0.5 in bands 13–108 is excluded.

The accuracies of land surface reflectance retrieval are evaluated in terms of the RMSE and RRMSE values between 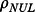 and 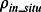 for targets H1–H4 and M1–M15 in bands 13–108. The results are shown in Figure 16A. The RMSE values range between approximately 0.01 and 0.07, while the R-RMSE values are between approximately 5% and 12%. The largest RMSE value occurs in the near-infrared region, while the smallest RMSE value occurs in the visible range. In contrast, the largest RRMSE value occurs in the visible region, while the smallest RRMSE value occurs in the near-infrared range. The accuracies of 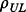 are similar to those of 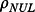, which are not shown in this study.

To further demonstrate the RMSE and RRMSE values between 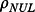 and 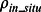 for the 19 targets in the linear response range of the UAV-HYPER sensor, surface reflectance greater than 0.5 in the bands 13–108 are discarded. The results are shown in Figure 16B. The RMSE values are between approximately 0.005 and 0.05, which is obviously smaller than those shown in Figure 16A. However, large RMSE values also occur in the near-infrared region. The RRMSE values are between approximately 4% and 10%, which is slightly less than those shown in Figure 16A.

### Conclusions

The radiometric performance of the UAV-HYPER sensor was assessed in terms of SNR and the accuracy of the radiometric calibration. The SNR values estimated using the homogeneous targets were between approximately 5 and 120. The linear response of the UAV-HYPER sensor was found in the apparent reflectance range of approximately 0.05 and 0.45, while a non-linear response was observed for apparent reflectance greater than approximately 0.45. The accuracies of the radiometric calibration of the UAV-HYPER sensor were evaluated with RMSE of approximately 0.01–0.06 and RRMSE of approximately 4%–10%.

The retrieved uniform Lambertian land surface reflectance match the *in situ* surface reflectance well in the land surface reflectance range of approximately 0.05 to 0.5. There is a small difference between the retrieved uniform and non-uniform Lambertian land surface reflectance over the homogeneous targets and under low AOD conditions. The results demonstrate that the discrepancy between the uniform and non-uniform Lambertian land surface reflectance can be neglected under homogeneous surface and low AOD conditions. However, the discrepancy is up to 0.08 in the blue region when adjacent pixels have large land surface reflectance contrast and under high AOD conditions (e.g. AOD = 1.0). Therefore, this discrepancy should be taken into account under these conditions. The accuracies of land surface reflectance retrieval were evaluated using the *in situ* measurements with RMSE of approximately 0.01–0.07 and RRMSE of approximately 5%–12%.
