# Correction: Land Surface Reflectance Retrieval from Hyperspectral Data Collected by an Unmanned Aerial Vehicle over the Baotou Test Site

**DOI:** 10.1371/annotation/09d10115-c53d-4d9f-8036-85c476eeef38

**Published:** 2013-08-01

**Authors:** Si-Bo Duan, Zhao-Liang Li, Bo-Hui Tang, Hua Wu, Lingling Ma, Enyu Zhao, Chuanrong Li

The order of affiliations for the corresponding author, Zhao-Liang Li, is incorrect. The correct order can be found below:

Zhao-Liang Li^4,3^

**4** Key Laboratory of Agri-informatics, Ministry of Agriculture/Institute of Agricultural Resources and Regional Planning, Chinese Academy of Agricultural Sciences, Beijing, China

**3** Laboratoire des sciences de l’ingenieur, de l’informatique et de l’imagerie, Université de Strasbourg, Centre National de la Recherche Scientifique, Illkirch, France

